# Alcohol policy framing in South Africa during the early stages of COVID-19: using extraordinary times to make an argument for a new normal

**DOI:** 10.1186/s12889-023-16512-y

**Published:** 2023-09-28

**Authors:** Andrew Bartlett, Matthew Lesch, Su Golder, Jim McCambridge

**Affiliations:** Department of Health Sciences, Seebohm Rowntree Building, University of York, Heslington, York YO10 5DD England

**Keywords:** Alcohol, Alcohol Industry, Policy, COVID-19, South Africa

## Abstract

**Introduction:**

Public health and alcohol industry actors compete to frame alcohol policy problems and solutions. Little is known about how sudden shifts in the political context provide moments for policy actors to re-frame alcohol-related issues. South Africa’s temporary bans on alcohol sales during the COVID-19 pandemic offered an opportunity to study this phenomenon.

**Methods:**

We identified Professor Charles Parry from the South African Medical Research Council as a key policy actor. Parry uses a Twitter account primarily to comment on alcohol-related issues in South Africa. We harvested his tweets posted from March 18 to August 31, 2020, coinciding with the first two alcohol sales bans. We conducted a thematic analysis of the tweets to understand how Parry framed alcohol policy evidence and issues during these ‘extraordinary times.’

**Results:**

Parry underlined the extent of alcohol-related harm during ‘normal times’ with scientific evidence and contested industry actors’ efforts to re-frame relevant evidence in a coherent and well-constructed argument. Parry used the temporary sales restrictions to highlight the magnitude of the health and social harms resulting from alcohol consumption, particularly trauma, rather than the COVID-19 transmission risks. Parry portrayed the sales ban as a policy learning opportunity (or ‘experiment’) for South Africa and beyond.

**Conclusions:**

Crisis conditions can provide new openings for public health (and industry) actors to make salient particular features of alcohol and alcohol policy evidence.

## Introduction

Crises can generate sudden and unexpected shifts in public policy agendas [1–3], requiring major policy change. The onset of the COVID-19 pandemic was one such example [4–6]. The World Health Organization (WHO) and prominent public health experts recommended the adoption of social distancing measures, including lockdowns and the closure of non-essential services [7–9]. The scope of policy restrictions varied enormously across the globe [10–13].

As countries began introducing lockdowns in March 2020, there was an accompanying surge in alcohol sales [14]. In April 2020, the WHO warned that alcohol consumption could promote risk-taking behaviour and recommended that access to alcohol be restricted [15]. Several countries, including South Africa, India, and Thailand, imposed temporary bans on alcohol sales [16]. Particularly where there are few restrictions on alcohol availability, alcohol poses a major burden on healthcare and emergency services [17–19]. Alcohol-related problems can contribute to about 20% of injury and 11.5% of non-injury emergency room presentations [17]. Alcohol consumption can also increase virus transmission through increased social mixing [20, 21]. Finally, addressing alcohol-related domestic violence took on greater importance during lockdowns [22–24].

Alcohol availability restrictions are a well-evidenced approach to reducing health and social harm [19]. Yet public health advocates must compete for policymakers’ and the public’s limited attention. Crises that put a spotlight on alcohol-related issues can create new opportunities for experts and advocates to champion particular policy solutions [25, 26]. Historical studies have identified how periods of crisis have provided the grounds for major, lasting changes in alcohol policies [27, 28].

Policy actors’ ability to build political support is often contingent on framing, or the strategic presentation of policy-relevant information [29–35]. In the context of alcohol policy, framing studies have largely focused on the alcohol industry actors and their capacity to frame policy debates [36–39]. The alcohol industry frames alcohol problems and solutions by focusing on the individual rather than the population, advocating for ineffective strategies that align with its interests, including public awareness campaigns. Compared to the ‘best buy’ population-level solutions endorsed by the WHO – using prices/taxes, restrictions on advertising, and restrictions on availability – to reduce harm through reducing population-level consumption, the strategies advocated by industry present little risk to revenue [37, 38, 40–43]. Yet public health actors can also re-frame issues to focus on evidence of the harm that alcohol poses to the population more broadly, and what can be done about it. These arguments are often important in securing evidence-informed policy change [31, 34]. Other studies show how identifying the range of harm that alcohol consumption causes – both to self and others and at an individual and a population level – can help enlarge the scope of public health coalitions [32]. Thus, it is now well known that how different policy actors portray alcohol-related issues and bring evidence into play can help shape debates about alcohol policy and the resulting decisions [38].

The purpose of this study is to examine how sudden shifts in the political context can create new opportunities for actors to frame alcohol-related policy evidence and issues. Focusing on the South African context, we investigate how a prominent public health scientist seized upon the temporary alcohol restrictions to re-frame alcohol policy issues, making consideration of the existing evidence base relevant in this novel context. Specifically, we examine the social media activity of Professor Charles Parry in the early stages of the pandemic. His Twitter account is used to examine how public health actors can use crises as windows of opportunity [3] to draw attention to the evidence on alcohol-related problems and the policy measures needed to promote public health.

## Methods

To conduct this study, we identified Professor Charles Parry as a key policy actor. Professor Parry is an epidemiologist and policy analyst. He is the Director of the Alcohol, Tobacco & Other Drug Research Unit at the South African Medical Research Council (SAMRC) and serves on several groups and committees, including the WHO Expert Panel on Drug Dependence and Alcohol Problems; the WHO Technical Advisory Group on Alcohol & Drug Epidemiology (Chair 2015–16); the board of the Global Alcohol Policy Alliance; and the UN Office on Drugs & Crime's World Drug Report Scientific Advisory Committee. 

Parry is a highly active Twitter user (@profparry), with approximately 1850 followers. His Twitter account is primarily used to identify and comment on alcohol-related issues in the South African context. For this study, SG harvested 3039 tweets posted between October 2017 and June 2021. The vast majority of these tweets address the topic of alcohol policy. We narrowed the focus of our analysis to tweets between March 18, 2020, and August 31, 2020, to coincide with the first two alcohol bans enacted in South Africa (see Table 1). The first ban lasted from March 27 to May 31, whilst the second ban was implemented between July 13 and August 17. These events were obviously important developments with high profiles internationally, providing moments where framing contests may be expected to occur. Our analysis therefore covered- tweets made during the period of the two bans as well as potentially relevant tweets that immediately preceded or followed these measures being enacted.

**Table 1:** Timeline of events

Date	Policy development
15 March 2020	President of South Africa declares the COVID-19 pandemic a “national disaster”
18 March 2020	First wave of COVID-19 regulations is introduced. This includes prohibiting gatherings of more than 100 people, and limits on the sale, dispensing and transportation of alcohol
27 March 2020	Level 5 COVID-19 regulations are implemented, including prohibiting the sale of alcohol
31 May 2020	The first ban on alcohol sales is lifted but some restrictions remain in place
13 July 2020	The ban on alcohol sales is re-implemented
18 August 2020	Second ban on alcohol sales is lifted
28 December 2020	Third ban is alcohol sales is introduced
2 February 2021	Third ban is alcohol sales is lifted

To analyse the data, AB and ML used thematic analysis [44]. Codes were developed using deductive approaches, drawing primarily from existing studies of alcohol policy [29–32, 34, 40] and inductive approaches, based on an initial reading of the tweets (see Table 2) following team discussions. Before coding the entire dataset, AB and ML independently coded a sample of 55 tweets to ensure there was consensus on the meaning and application of codes. AB completed coding the remaining tweets (343). In total, 398 tweets were coded. The final analysis, which subsequently involved the development and refinement of the thematic content by the team as a whole, is reported here.

**Table 2:** Coding of Parry’s tweets

Code	Description	# Tweets
Alcohol-Related Harm	in which alcohol-related harm (aside from covid transmission) is discussed	221
Accidents	in which the contribution of alcohol consumption to accidents is discussed	45
Violence	in which the contribution of alcohol to violence is discussed	54
Gender-Based Violence	in which gender-based and domestic violence is specifically discussed	28
Loosening of Restrictions	in which the loosening of restrictions is discussed	164
Measures of Effectiveness	in which evidence of the effects and efficacy of changes in regulation is discussed	124
Industry as an Actor	in which the industry is identified as an agent/actor	116
Burden on the Healthcare System	in which alcohol-related harm is framed as a burden on the health care system/resources	68
Post-Covid	in which alcohol regulations and alcohol harm post-covid restrictions are considered	66
Covid Transmission	in which the effect of alcohol regulations on covid transmission is discussed	44
Addiction and Dependence	in which the effects of covid-related alcohol regulations on addiction and dependence are discussed	24
Essential Commodity	in which the idea of alcohol as an essential commodity/service is discussed	11

In the text, explicit reference to the codes used presents the name of the code in small caps

Denotes a sub-code

By focusing on one expert’s day-to-day contemporaneous reflections on and reactions to developing conditions we are drawing on a particularly rich body of data; a veritable ‘Journal of the Plague Year’. This is not a study of the political impact of Parry’s Twitter activity. Twitter, in this case, is not the object under study, but is an accessible source of Parry’s ‘framing’ providing the basis for an in-depth study.

We consider how Parry’s tweets can be understood, collectively, as an *argument*. This argument has a logic and a structure, and by understanding that we can distil the function of Parry’s framing efforts [45]. Note that while readers may infer *intent* and speculate on the extent to which Parry’s tweets in August were a direct continuation of the logic of his tweets in March, for example, this paper can only access the textual traces Parry has left on Twitter. For the purposes of this paper, the coherence of Parry’s argument across this body of tweets is emergent. The pandemic offered an opportunity to present this argument, not so much because it was an unprecedented public health crisis in itself, but because it afforded a new perspective on alcohol-related-harms during ‘normal’ as well as ‘extraordinary’ times.

## Results

### Policy context and Parry’s orientation

The South African government enacted a comprehensive public health response to the COVID-19 pandemic, including several nationwide lockdowns. The legal basis for these restrictions was the 2002 South African Disaster Management Act (DMA). The government’s efforts to curb the spread of the virus included travel bans as well as outlawing large social gatherings. Businesses were only able to operate if they were engaged in “manufacturing, supply, or provision of an essential good or service.” The DMA also permitted the government to temporarily ban “the sale, dispensing or transportation of alcoholic beverages” [46].

On March 26 2020, these specific alcohol provisions were enacted, with businesses that provided alcohol for consumption on and off premises ordered to stop selling alcohol. The main rationales for this were to reduce social gatherings and thus reduce transmission, and to limit the number of alcohol-related trauma cases to free up resources for COVID-19-related care [47]. The government adjusted alcohol regulations in response to the rate of COVID-19 infections. When COVID-19 cases were rising, the government prohibited the sale of alcohol, and when they were declining these were eased, with restrictions on the days and times during which alcohol sales would be permitted (see Table 1, 24 May 2020) [47].

Alcohol sales bans were particularly significant given the political strength of the alcohol industry in South Africa and the failure of pre-pandemic efforts to implement other population-level alcohol policy changes [48, 49]. In 2013, alcohol industry actors lobbied against the Control of Marketing of Alcoholic Beverages Bill and the legislation was never implemented [50]. During COVID-19 restrictions, alcohol industry actors emphasised negative economic impacts, illegal trade, and limited evidence on the effectiveness of alcohol sale bans [47].

Alcohol is widely seen as a major public health problem in South Africa, contributing to all forms of violence, injuries, trauma presentations, and premature mortality [48, 49]. The World Health Organisation (WHO) ranks South Africa as the country with the third highest per capita alcohol consumption in Africa, behind only its neighbours Namibia and Eswatini [51]. A 2012 comparative study identified South Africa as the African nation with the highest alcohol-attributable burden of disease [52]. Indeed, in 2012, “alcohol-attributable harm accounted for an estimated 7.1% […] of all deaths” in South Africa, with alcohol’s contribution to the transmission of TB and HIV/AIDS, to road traffic accidents and interpersonal violence especially notable [53]. The connections between alcohol and violence might help explain why there was public support for alcohol restrictions. For example, during the first ban, in a poll conducted in April 2020 by the University of Johannesburg, only 12% of South Africans supported removing the restrictions on the sale of alcohol [54]. Moreover, as the Social Development Minister explained, the ban on alcohol would allow South Africa “to examine issues that have been vexing […] this country for a very, very long time” [55].

Relatively few of Parry’s tweets focused on the potential for alcohol consumption to contribute to covid transmission (*n* = 44). The main focus of Parry’s tweets was on alcohol-related harm (*n* = 221), with Parry typically using “trauma” as a shorthand way to describe intoxication-related alcohol harm, with the word ‘trauma’ appearing 153 times. In fact, ‘trauma’ is the third most commonly used word in his tweets after ‘alcohol’ and ‘liquor’, ahead of ‘#covid-19’ and ‘alcohol-related’. Before the pandemic, however, the term ‘trauma’ was not used. In some instances, the sources of this trauma were broken down into accidents (45 tweets) and violence (54 tweets). Over half (*n* = 28) of the tweets coded for violence specifically identify the politically salient issue of gender-based violence [56].

Concerns about the burden on the healthcare system were central to the government’s argument for temporarily banning alcohol sales, and Parry’s tweets reflected this. However, tweets coded burden on the healthcare system (*n* = 68) accounted for only a relatively small proportion (approximately one-third) of overall tweets coded alcohol-related harm.

For example, prior to the initial alcohol ban commencing, Parry wrote:

*Thu Mar 19 2020*

“[it] will be interesting to note level of decrease in injuries and deaths from interpersonal violence from new emergency regulations regarding liquor sales in South Africa, but confounders will be hard to control. #socialisolation #COVID19 #Covid19SA”

Here, Parry is anticipating how the restrictions could help the public, as well as policy participants such as public health and government officials, better understand particular alcohol-related harms and the ways in which they may be reduced.

Parry’s tweets also helped reinforce the government’s prevailing policy argument about the need to reduce the burden on the healthcare system. For example, when South Africa announced the easing of alcohol restrictions, he shared the following:

*Fri May 29 2020*

“Cape Town faces a dire ICU bed shortage. Just imagine what it is going to be like after ~10,000 liquor outlets start trading in province next Monday for 32 hours/week. Life & death choices will have to be made: #Covid19 vs trauma patients”

Nevertheless, most of the tweets coded for alcohol-related harm did not directly discuss trauma as an obstacle to treating COVID-19 patients but rather discussed the harms of alcohol *on their own terms*. Identifying the risks or harms of alcohol that are a result of the interaction between alcohol and COVID-19 – either by increasing transmission or by placing a burden on the healthcare system – is fundamentally different from discussing the harms of alcohol themselves, which was what Parry was also doing.

### Crisis as experiment

Parry’s tweets focusing on alcohol-related harms irrespective of the COVID-19 crisis established a ‘baseline.’ In the next step of his “argument”, Parry’s tweets focused less on whether the government had successfully mitigated the impact of the *pandemic* and more on how the different alcohol restrictions affected levels of *alcohol-related harms* during these ‘extraordinary’ times. As such, Parry’s core argument held important implications for alcohol consumption and associated harms during ‘normal’ times as well.

One hundred twenty-four of Parry’s tweets during this period were coded for measures of effectiveness; tweets in which he discussed the evidence that the various stages of lockdowns and subsequent loosening of restrictions had had demonstrable effects. 75% (*n* = 93) of these tweets were also coded for alcohol-related harm, providing evidence that COVID-19 outcomes were not the principal focus. As early as late March, Parry identified the effectiveness of alcohol restrictions in reducing trauma (at this point, the full ban on sales had yet to be imposed):

*Fri Mar 27 2020*

“Preliminary data show initial #COVID19 regulations mandating earlier closing times for on- and off-consumption liquor outlets in South Africa has a positive impact.”

As data became available, Parry’s tweets cited statistics to illustrate significant shifts in alcohol-related harm and to invite thinking about the effects of easing the restrictions on alcohol. For example, in April, during the first lockdown, Parry tweeted:

*Tue Apr 14 2020*

“SA has ~1.5 million trauma admissions/yr. ~1/3 are alcohol-related (~10,000/week). Big drop in alcohol-related trauma admissions from #LockdownSA, possibly 7,000/week. If limited alcohol sales opened up we might see ~1,000 cases/week returning to trauma units. Is this acceptable?”

Parry thus used this moment to ask his audience whether the level of alcohol-related harm existing pre-crisis and anticipated post-crisis were ‘acceptable’, thus framing the problem shaped by a contrast between differing levels of harm experienced in different policy contexts.

In May, Parry shared data indicating the favourable consequences of the South African approach internationally:

*Fri May 29 2020*

“South Africa is one of two countries reported in a (UK) Financial Times article yesterday where ‘excess’ deaths at this time are in the negative -- probably due to the early, hard lockdown which also included major restrictions on alcohol availability”

The ‘experiment’ ran in both directions; after a period in which the taps were turned off the alcohol flow [57] began to be restored. At the start of June, Parry used his Twitter feed to report on the increases in trauma presentations:

*Thu Jun 04 2020*

“With South Africa now experiencing a rapid increase in alcohol-related trauma & non-natural deaths again in #lockdown3 following lifting of ban on alcohol sales without additional countermeasures besides limiting sales to 8 hours on 4 days it is time to read this report again”

Parry is responding to a WHO Europe tweet about population-level increases in life expectancy associated with a decrease in alcohol consumption, based on work done in Russia. Similarly, just over a week later, Parry underlines findings reported by eNCA (a Southern African 24-h news channel):

*Fri Jun 12 2020*

“Looks like a 70.4% increase [((685-402)/402)*100] in trauma from 5 Western Cape hospitals from last week Level 4 to 1st week Level 3. Many alcohol-related. Supports our projection of 5,000 ↑ in alcohol-related trauma admissions/week nationally.”

Parry’s tweet is one of several that not only addresses the trauma associated with alcohol consumption but also the *scientific* work that he was leading at SAMRC modelling the impacts of the policy changes. He was using Twitter to communicate these findings. This work began early in the crisis. For example, in April, Parry tweeted:

*Wed Apr 15 2020*

“A reworking of our model on the effect of permitting off-consumption liquor sales following challenges to #COVID19 #lockdown ban on sale of alcohol in South Africa from 27 March shows that this could result in >4900 new alcohol-related admissions to trauma units/week in SA”

Parry was engaging with the evidence produced in this extraordinary moment, by his team and others, making public interventions to improve the public understanding of science with regard to discussions around alcohol policy issues.

### Industry as opponent

Parry focused specifically on the alcohol industry’s role in alcohol-related harms. The alcohol industry makes and markets alcohol, shapes legislation around alcohol sale and consumption, and participates – contentiously – in the scientific study of alcohol harms [38, 40, 58, 59]. industry as an actor was coded for 116 tweets. Some of the tweets included industry as an actor is positioned as a *subject* of regulation, such as when Parry calls for restrictions on alcohol advertising. Parry also directly engaged with industry actors and/or their arguments on Twitter and monitored the alcohol industry’s activities, providing additional context for interpreting the industry’s actions.

Parry presented South Africa’s alcohol industry as a self-interested actor whose material interests are in direct conflict with public health. For example, during the first lockdown, a leading alcohol industry association, the Gauteng Liquor Forum, threatened to take legal action against the government. In response, Parry asked:

*Mon Apr 13 2020*

“Is this a case of putting ‘profits before people’ and the narrow interests of one sector above those of the entire country? #covid19 #LockdownSA @BigAexposed”

Parry’s tweet is a political, not scientific, intervention; as it does not turn on the interpretation of evidence or other knowledge claims, but on a question of policy *priorities*.

Second, Parry offered highly critical commentary on the alcohol industry’s rhetoric, which does draw on scientific evidence, including the industry’s commitment to promoting “responsible drinking.” In mid-winter (in the Southern Hemisphere), during the second alcohol ban, Parry took aim at the major companies:

*Thu Jul 30 2020*

“#bigalcohol is making a lot of noise about current sales ban while motivating for “responsible drinking”. There is no way that nationwide moderate drinking would come close to supporting continuation of the industry as it currently exists. Impact on sales would be catastrophic”

Third, Parry identified public relations strategies used by the alcohol industry to challenge the ban on alcohol sales. In one tweet, Parry referenced an article written in New24.com, which described the alcohol ban as “unprecedented and misguided” [60]. Parry highlighted that the article’s author had a history of lobbying for the tobacco industry:

*Sun Jun 21 2020*

“The @UniofBath's Tobacco Tactics unit details @BigTobacco links of the Consumer Choice Center whose Fred Roeder wrote an opinion piece in @news24 today, ""Cigarette and alcohol bans are unprecedented and misguided"". @AdriaanBasson.”

In another instance, Parry questioned the motives, credibility, and credentials of another alcohol policy opponent, who had been granted a platform in the news media. Marjana Martinic, a consultant to, and former high-level employee of global public relations (PR) organisations of the major alcohol companies appeared in various South African media outlets to voice opposition to the restrictions. For example, in one article, Martinic argued “[h]owever well-intentioned the South African […] lockdown poses a particular risk to health, stokes organised crime and deprives the fiscus[sic] of significant revenue at a time when it is needed most.” Parry underlined Martinic’s extensive history with the alcohol industry. For example,

*Fri May 15 2020*

“What [the] article fails to add is that the "international expert" [Marjana Martinic] has <5 peer reviewed publication in Scopus, an h-index of only 3 & worked for a liquor industry front organisation (ICAP now IARD) for 21 years.”

In another, tweet Parry continued:

*Fri May 15 2020*

“it will be important to ask if her consultancy firm is being paid by @SABreweries or the industry front, International Alliance for Responsible Drinking, to do interviews and write articles against the lockdown on liquor sales in South Africa”

Martinic and News24.com failed to disclose transparently any of her associations with the alcohol industry.

Parry monitored other alcohol industry actors and their claims put forward on social media. During the summer, a global PR company commissioned a YouGov poll on public attitudes toward the alcohol restrictions in South Africa. IARD tweeted that “almost half of the respondents […] said that their country’s restrictions on alcohol sales during COVID-19 shutdown were too strict.” Parry responded to the IARD, putting those polling results into perspective, saying:

*Fri Jun 12 2020*

“ .. but 54% did not agree that the alcohol restrictions in South Africa during #lockDownSouthAfrica were too strict. Thanks for making this clear @VictimOfMaths. Note that IARD is a front organization for major global alcohol producers.”

Again, Parry makes clear how the alcohol industry uses front groups, like IARD, for public relations purposes.

As the evidence of the effectiveness of South Africa’s restrictions on reducing the harm produced by alcohol began to emerge, Parry’s engagement on Twitter with industry actors adapted in turn as he became engaged in disputes in the technical domain [61]. Many of the tweets coded for industry as an actor and measures of effectiveness concern a debate over the policy implications of the lockdown restrictions evidence.

Parry was critical of how alcohol industry actors were using social media to present misleading information on the effects of the restrictions. In August, SAB (South African Breweries, now part of the world’s largest brewer, ABInBev) tweeted an image of a press release arguing against a “suspension of alcohol and beer sales”, claiming that prior restrictions had led drinkers to turn to illegal alcohol with “dire public health consequences.” The tweet also included the hashtag #ResponsibleTogether. Parry was highly critical of these arguments and responded when they were posted. In responding to a News24.com article headlined, “Alcohol sales ban cost drinks giant Distell nearly 20% of its trading year, says CEO” [62], Parry tweeted:

*Thu Aug 27 2020*

“If illegal sources of alcohol had been substantial during bans on alcohol sales we would not have seen such a dramatic drop in trauma presentations, GBV, and non-natural deaths. Can Ruston [CEO of Distell] provide evidence to back up his claims of massive illegal sales?”

There were also industry attacks on the models that Parry and others have used to evaluate the impact of the bans. In one news article, Sibani Mngadi, Corporate Relations Director of Diageo in South Africa, claimed that the data used by the government “to justify the ban on liquor sales didn't include hospitals built in the past 20 years.” Parry challenged the disinformation directly by asking:

*Fri Aug 07 2020*

Why is Sibani Mngadi still saying that modeling behind liquor ban did not take into account new hospitals built when we have stated that is not the case? Saying something not true over & over again does not make something true. #factsmatter

Some of the alcohol industry’s allies, including those working in the press, echoed this argument about poor data quality. For example, the Financial Mail published an article headlined: “Dlamini Zuma relied on ‘unsound scientific data and hearsay’”(referring to Nkosazana Dlamini-Zuma, the minister implementing most of the COVID restrictions) [63]. Parry responded by saying:

*Fri Aug 14 2020*

“'Unsound scientific' data has accurately predicted ↓ in trauma presentations based on data from 10 hospitals to date. No one held view that ALL (or even majority) of trauma reductions were due to liquor sales ban rather than other restrictions on movement ('Straw Man Fallacy')”

For Parry, engaging with industry actors during the COVID-19 crisis was not simply about highlighting their role in alcohol harm, but defending public health research from their “merchants of doubt” [64].

*Sat Jun 06 2020*

“I doubt we will hear the truth from liquor industry, but the massive burden from alcohol has been exposed by ban on alcohol sales in South Africa. We can't unsee what we have seen.”

### Towards a new normal with effective regulation, not prohibition

Parry used the crisis as an opportunity to promote the idea of a new normal, and to offer ways for South Africa to manage alcohol differently after the crisis passed. There were 66 tweets coded for post-covid, showing that imagining the future differently figured prominently. Parry, even from early in the crisis, is clear that this future is decidedly not to be a continuation of a ‘prohibition’. Rather, the end goal is a re-opening of bars and restaurants, a return to the sale and consumption of alcohol, but under new terms. For example, in April when Max du Preez, a veteran South African journalist with nearly 300 k followers on Twitter, asked whether it would be “feasible to enforce this ban for 6 months or more?” Parry responded by saying:

*Mon Apr 27 2020*

“We only need to get to Level 3 to have alcohol sales resume. I really hope we can get to #lockdown level 3 in a few months. We must try! I don't support a long-term ban on alcohol sales & it is really only defendable as a curb to community transmission & if we need hospital beds”

It should be noted that while Parry was seemingly content to use the word ‘prohibition’ at some points and for clear purposes, he would respond when the word was weaponised in a pejorative sense. In one exchange, another user claims that public health actors in South Africa “haven’t learned nothing from US prohibition or the War on Drugs.” In response, Parry states:

*Sun Jul 12 2020*

“Prohibition means you can't drink alcohol or even brew your own. What we have is a temporary sales ban to save lives. You obviously haven't read my paper, ""Beyond the rhetoric: Moving towards a more effective and humane drug policy framework in South Africa" S Afr Med J 2011/101”

Later in the same month, Parry was challenged to provide evidence that ‘prohibition’ works, to which he replies:

*Tue Jul 28 2020*

“I can't. Fortunately we don't have prohibition in SA, we have a temporary sales ban. This is very different from the failed US policy. No one is prohibited from consuming alcohol or even brewing their own, just from selling to others or purchasing it.”

These two examples are occasions in which Parry engaged with members of the public, in exchanges founded on the alcohol industry’s efforts to frame South Africa’s restrictions this way. For example, responding to a SAB tweet promoting a webinar with “international experts discuss[ing] the impact of prohibition of alcohol” [65] in the South African context, Parry tweeted:

*Sat Aug 01 2020*

“Not sure how useful it will be to discuss ‘prohibition’ when what South Africa has is a temporary sales ban. Alcohol is not prohibited, just the sale thereof. Hopefully that will soon be lifted, but with stronger controls on availability, marketing, products, & drunk driving”

Again, it should be noted that, during the early days of the crisis, Parry used the word ‘prohibition’ to describe what was happening in South Africa. Historically, in some cases, ‘prohibition’ has meant a ban on sales, but not necessarily on consumption. In these tweets, however, we see how Parry’s opposition to the industry’s ‘prohibition’ framing is connected to his imagining of a ‘new normal’, a future in which South Africa has “stronger controls on availability, marketing, products, & drunk driving”. Parry avoids the ‘prohibition’ framing for the same reason it is a powerful way to frame arguments against regulation; it carries with it a spectre of authoritarian permanence. Effective alcohol policy measures that work by reducing overall consumption have long been framed as ‘prohibitionist’ by industry, seeking to associate these policies with an emphasised failure of the U.S. national prohibition [39, 66], and Parry’s tweets push back against this conflation.

From early in the crisis, Parry anticipated a post-covid future in which South Africa had a different relationship with alcohol. In April, City Press published an article headlined, “Bheki Cele: ‘I wish alcohol ban could be extended beyond lockdown’.” Parry responded to the story by saying:

*Mon Apr 06 2020*

“@City_Press The ban on alcohol sales will be lifted post #lockdown, but we certainly should do more to reduce the ~172 deaths/day from alcohol-related causes in South Africa & should not return to business-as-usual for #bigalcoholexposed vis-a-vs alcohol post #covid19 #lockdownSouthAfrica”

By using the hashtag #bigalcoholexposed, Parry was attributing responsibility for alcohol-related harm to the alcohol industry and anticipating the industry’s role as an obstacle to any new normal. For Parry, the alcohol *industry*, more so than alcohol itself, is the key object of regulation. This is made clear when Parry interacts with a SAB tweet (hashtagged #LiftheBan and #ResponsibleTogether) which warned against repeating old mistakes “made 100 years ago”, evoking memories of US prohibition. Parry responded by saying:

*Sat Aug 15 2020*

“Mistake we have made in SA is failure to properly regulate alcohol industry. A ban on sales is not a long term solution. We need to properly regulate & enforce alcohol availability, marketing, harmful products, drink driving laws & have effective pricing & labeling policies”

That the crisis – and the government’s response – is an ‘opportunity’ is a common theme throughout Parry’s tweets. For example, in April, responding to a tweet from, the Chief Economist of the Agricultural Business Chamber of South Africa, Parry wrote:

*Mon Apr 13 2020*

“Level of dependency of South Africans on booze & cigarettes is staggering as is fact that alcohol & cigarette industries are reliant on this such dependent consumers for their profits. Is #covid-19 #LockdownSA an opportunity to break this symbiotic relationship? @DrZweliMkhize”

Parry also tagged in Dr Zweli Mkhize, who at that time was the Minister of Health. The opportunity presented by the crisis lay in the lessons that could be learned regarding effective alcohol regulation. For example, in June, Parry concludes:

*Sat Jun 13 2020*

“What #lockDownSouthAfrica & the 8 day period of #lockdownlite, the 66 day ban on alcohol sales in L4&5, & subsequent lifting of ban in L3 has revealed is the need for an urgent national effort to address heavy drinking. We should not miss the opportunity to create a new normal”

This is expressly about the *evidence* provided by the emergency regulation, and the way this can be used to reshape regulation outside of the moment of crisis. Similarly, in August, tweeting a link to a story on Independent Online (IOL), headlined, “How to ensure an ‘alcohol safe’ SA”, he wrote:

*Mon Aug 17 2020*

According to @Saapa7: “South Africa’s pre-existing challenges with alcohol-related harm have been highlighted by Covid-19. It’s an opportunity for government and society to establish a new and better ‘normal’ in our relationship with alcohol...”

He is quoting the Southern African Alcohol Policy Alliance from the article, but this positions alcohol harm as something that ‘pre-existed’, a feature of ‘normal’ times, the scale of which, and potential regulatory solutions to, were revealed by the crisis.

## Discussion

This is a study of issue framing in a highly politically charged context, focusing on the activities of one significant scientific figure and their alcohol policy evidence entrepreneurship. Using this accessible, day-to-day record of responses and reactions to developing conditions, this paper is a study of the how Charles Parry, a leading public health expert, used the extraordinary times of the onset of the COVID-19 pandemic, and the subsequent alcohol sales restrictions in South Africa, to invite thinking about the acceptability of high levels of alcohol harm in normal times. Parry drew attention to well-established evidence on alcohol harm, and new evidence on the effectiveness of restrictions in reducing alcohol-related harms. These linkages, skilfully made, were in part premised on the well-established relationship between alcohol and violence in South Africa and the disruption of the relationship made possible by the temporary alcohol policy measures. The crisis conditions themselves, and in turn the policy responses, thus invited new contexts for the framing contest between public health actors and the alcohol industry. Since public health considerations provided the dominant frame for policy measures, public health scientists could be more assertive, whilst the industry found itself on the defensive. This all made for a moment in which the political decision-making about the enactment of measures in response to COVID-19 was “up for grabs”, affording opportunities for public health policy evidence entrepreneurship [3, 67, 68] which were seized by Parry. This is consistent with previous research that shows the importance of “coupling” [3] policy problems with solutions during fleeting windows of opportunity [25, 31, 32, 40].

This paper is not a study of the political impact of Parry, but of how the conditions produced by the COVID-19 crisis provided an opportunity to reframe the debate around the harms of alcohol. That Parry had just under two thousand followers is neither here nor there. For the purposes of this paper Twitter is not in itself the forum in which political understanding of the harms of alcohol is re/made, here merely a data source that permits in-depth investigation, of the developing thinking of a significant scientific figure in dialogue with fast- changing policy-related events.

Parry’s emphasis on aggregate population-level harms is significant. This allowed the argument for regulation as the dominant frame for thinking about future policy, using this moment of crisis to propose that society can now decide to choose a new normal. Making this argument required taking on industry actors’ conflation of known effective whole-population policy measures with prohibition. It also involved using the crisis to present the industry, and its arguments, as being ‘exposed’, and portraying the industry as part of the problem. Parry’s tweets in the South African case further underscore the inter-connectedness of the framings of problem, solutions and actors and the central role of public health actors in making those linkages. Parry’s promotion of empirical data as it became available was another feature of his approach, making for a strong contrast to the alcohol industry’s handling of data in its argument.

Strengths of this study include the identification of an example of the knitting together of problem, solution and actor frames. In the case of South Africa, these frames were not only developed in the agenda-setting phase of policy making [69] but extended over multiple iterations of policy change induced by the crisis conditions. The dynamic nature of the contest, and the counterposing of scientific data with rhetoric, are captured well in this dataset. Twitter thus may be an important data source for future studies of framing contests [70–72].

Study limitations should also be borne in mind. The dataset comprises the tweets of a single actor, and any effects the tweets had on the unfolding of events is unclear; this is not a study of South African alcohol policy [47, 48] or framing effects [73] but a study of framing in the context of alcohol policy [29–31, 34]. Although this data is a rich source, providing researchers with access to the ideas and arguments of different interest groups, a more ambitious undertaking is needed to gain insights into multiple actors, including those operating within decision-making roles. Few countries adopted measures similar to those in South Africa to combat COVID-19, so careful attention to context, culture and history is needed to consider the generalizability of the key findings on framing. In this, the alcohol policy response to COVID-19 echoes the variety of ‘prohibitionist’ policies that arose out of the First World War and other crises of the early C20th [27]. The particular conditions of South Africa, particularly the political salience of violence, allowed the Government to justify more wide-ranging restrictions on alcohol sale and consumption than found in other countries. In South Africa, this was always intended to be a temporary policy response.

The research implications of these findings are straightforward. Alcohol policy researchers have traditionally studied framing by examining media coverage [74–76]. Yet public health issues, including alcohol-related harm, are increasingly being debated over social media, particularly Twitter [72, 77]. We require a deeper understanding of how these shifts in political communication are influencing policy actors’ capacity to frame scientific evidence and challenge competing frames. Beyond this, we need further studies that explore how key scientific and policy actors frame and have framed alcohol issues in different national and international alcohol policy contexts. Contemporary studies of this nature will help develop further understanding of key influences on the processes of alcohol policymaking and their outcomes. Such data can then be analysed comparatively across the world and in connection with how public health interests compete with other unhealthy commodity industries. Such studies may carry implications for alcohol policy evidence entrepreneurship more broadly.

## Data Availability

The paper used publicly available data.
